# Bereaved family member perceptions of patient-focused family-centred care during the last 30 days of life using a mortality follow-back survey: does location matter?

**DOI:** 10.1186/1472-684X-13-25

**Published:** 2014-05-14

**Authors:** Fred Burge, Beverley Lawson, Grace Johnston, Yukiko Asada, Paul F McIntyre, Eva Grunfeld, Gordon Flowerdew

**Affiliations:** 1 Department of Family Medicine, Dalhousie University, 5909 Veterans Memorial Lane, Abbie J. Lane Building, 8th Floor, Halifax, NS B3H 2E2, Canada; 2 School of Health Administration, Dalhousie University, 5161 George St, Suite 700, PO Box 15000, Halifax, Nova Scotia B3H 4R2, Canada; 3 Community Health and Epidemiology, Dalhousie University, Center for Clinical Research (2nd & 4th Floors), 5790 University Avenue, PO Box 15000, Halifax, Nova Scotia B3H 4R2, Canada; 4 Division of Palliative Medicine, Department of Medicine, Room 307, Bethune Building, Queen Elizabeth II Health Sciences Centre, 1276 South Park Street, Halifax, Nova Scotia B3H 2Y9, Canada; 5 Department of Family and Community Medicine and Ontario Institute for Cancer Research, University of Toronto, 500 University Avenue, Room 352, Toronto, Ontario M5G 1V7, Canada

**Keywords:** Terminal care, Caregivers, Health care surveys

## Abstract

**Background:**

Improving end-of-life care is an important international issue. Recently Nova Scotia researchers conducted a mortality follow-back survey to provide a population-based description of care provided to adults during their last 30 days of life as perceived by knowledgeable bereaved family members. Here we describe the relationship between the location where the decedent received the majority of care during their last 30 days and the informant’s perception of the extent of unmet need, as defined by multiple domains of patient-focused, family-centred care.

**Method:**

Death certificate identified informants (next-of-kin) of eligible adults who died between June 2009 and May 2011, in Nova Scotia, Canada were invited to participate in a telephone interview based on the After-Death Bereaved Family Member Interview. Whether or not the informant expressed unmet need or concerns for six patient-focused, family-centred care domains were assessed in relation to the location where the majority of care occurred during the decedent’s last 30 days.

**Results:**

1358 informants took part (25% response rate). Results of 1316 eligible interviews indicated home (39%) was the most common location of care, followed by long-term care (29%), hospital (23%) and hospital-based palliative-care units (9%). Unmet need ranged from 5.6% for dyspnea help to 66% for the emotional and spiritual needs of the family. Although the mean score for overall satisfaction was high (mean = 8.7 in 1–10 scale; SD 1.8), 57% were not completely satisfied. Compared to home, adjusted results indicated greater dissatisfaction with overall care and greater communication concerns in the hospital. Greater unmet need occurred at home for dyspnea. Less overall dissatisfaction and unmet need were expressed about care provided in long-term care facilities and hospital-based palliative-care units.

**Conclusion:**

Bereaved informants were generally highly satisfied with the decedent's care during their last 30 days but variations were evident. Overall, no one location stood out as exceptionally different in terms of perceived unmet need within each of the patient-focused, family-centred care domains. Communication in various forms and family emotional and spiritual support were consistently viewed as lacking in all locations and identified as targeted areas for impacting quality care at end of life.

## Background

Improving care for those at the end of life is quickly becoming an important issue among many countries. This is particularly true for countries such as Canada, where an aging population is signaling the need to provide care for more people with prolonged experiences of multiple chronic diseases as death approaches. Between now and the year 2056 the number of people who die in Canada will double to 500,000 per year [1]. Of these deaths, 80% are caused by end stage chronic diseases [2]. This aging demography and high prevalence of chronic disease will challenge healthcare systems around the world to provide good end-of-life care.

Although our knowledge about health service utilization at the end of life of Canadians who die with cancer is improving, we have much more to learn [3-5]. Health service utilization varies by population characteristics and despite a publically funded healthcare system, some inequalities are apparent. Whether or not these variations could be accounted for by patient need or preferences was not known as we had no population-based measures of these indicators, particularly of preferences. Moreover, we have almost no knowledge of these issues in Canada for those who died of advanced diseases other than cancer. Making the transition to a better designed and integrated, cross sector palliative approach to care for patients with advanced chronic disease is urgently needed [6].

Research on the dying experience cannot be carried out from the viewpoint of comprehensive Palliative Care Programs exclusively. Focusing on palliative care program patients only would result in selection bias [7] where the perspective on care provided is based on the “best” of our clinical care teams for the dying and, perhaps, on some of the most complex patients. For instance, in Nova Scotia 90% of patients in the largest palliative care program have a cancer diagnosis [6,8], yet cancer accounts for only about 30% of the deaths in the province [2]. We need to also examine all locations where end-of-life care is typically provided, such as home, hospital and long-term care settings.

Conducting a prospective cohort study recruiting patients “likely to die” is problematic given the imprecision of predicting death, the burden on the dying to respond to surveys and the costs associated with such a study [9]. One research approach using a population-based perspective is to obtain the views of proxies for the decedents using a key informant, such as a family caregiver, to give “voice” to the decedent’s experience of care. Studies using this approach have been published from the United States [10], the United Kingdom (UK) [11,12], Italy [13,14] and the Netherlands [15]. Most recently the first UK National Bereavement Survey (VOICES) 2011 [16] results were reported as well as a Netherlands study of older adults at the end of life [15]. These surveys have all used similar methods to ask bereaved family members or someone close to the decedent relatively soon after the death about multiple domains of patient-focused, family-centred care that the decedent received. Sampling frames were constructed using population-based approaches rather than identifying possible respondents from clinical program registry data. With few exceptions [10] results have been specific to the actual location of the death (e.g. hospitals) as opposed to location of care.

Canada lacked data that were population-based across all chronic disease causes of death, focussed on the locations of care (rather than the actual location of death), were sufficiently scaled to compare the location specific results and able to permit statistical adjustment in multivariable analyses to permit more rigorous interpretation of the results. Thus the goal of our study was to provide a population-based description of care provided to adults who died in Nova Scotia during the last 30 days of life as perceived by knowledgeable bereaved family members or informal caregivers (informant) shortly after death. In this article our objective is to describe the results of our survey process and to examine the relationship between the location where the decedent received the majority of care during their last 30 days of life and the informant’s perception of the extent of unmet need experienced, as defined by multiple domains of patient-focused, family-centred care [10,17], before and after adjustments for potentially confounding factors.

## Methods

### Design/setting

A mortality follow-back survey design was used to gather information about the experience of care provided to adults who died in the province of Nova Scotia, Canada, during their last 30 days of life, as perceived by a knowledgeable informant. Nova Scotia is a small, eastern province (population 950,000) with the highest proportion of residents aged 65 years and older in Canada [18] and with one of the highest population rates of cancer and diabetes [19,20]. Physician, hospital and homecare services are government supported as are medications for those 65 years of age and older. Long-term care (nursing home) residency is subsidized for those with insufficient income. Ethical approval for this research was provided by the Capital Health Research Ethics Board, Halifax Nova Scotia.

### Participants

Eligible participants (informants) were bereaved family members or informal caregivers knowledgeable about the medical care provided to an adult decedent during their last 30 days of life. Potential informants were identified using the ‘informant’ field (i.e., next-of-kin) on the death certificate of all adults (aged 18 years and older) who had died over a two-year period (June 1, 2009 – May 31, 2011) and available as confirmed deaths to the Nova Scotia Vital Statistics (NSVS) office at the time of selection. A maximal population [21] was initially identified which excluded records of decedents where the cause of death International Classification of Disease (ICD) codes were associated with an external cause or medical and surgical complications. Also excluded were death certificates where informant information was missing or incomplete. To ensure privacy and confidentiality, this identification and exclusion process was performed by NSVS staff who were provided a list of ICD codes for exclusion by the research team.

Additional exclusions included a limited number of death certificates associated with informants who had no knowledge of the medical care provided to the decedent and death certificates of those where the informant reported the decedent died very suddenly and had received no medical care of any kind during the 30 days prior to death. These latter exclusions could only be assessed if the informant contacted the researchers following the invitation to participate. In each of these situations the informant would not have been able to respond to questions asking about patient-focused family-centred care provided to the decedent. We acknowledge there are challenges and limitations associated with these exclusions [22].

Potentially eligible death certificates were identified every four months, over a 24-month period, for a total of six ‘waves’. Death certificates included in each wave for informant identification were limited to deaths occurring three to seven months prior to the start date of the wave, and to records available in the NSVS database at that time. This strategy was used to promote completion of each survey interview within ten months of the decedent’s date of death in order to maximize response among the bereaved [14] and to facilitate recall by providing a consistent yet relatively short and acceptable period of time [23,24].

### Procedure

A letter originating from NSVS was mailed to each potentially eligible informant inviting their participation and included study information and a choice of three ways (mail, toll-free telephone, email) to contact the researchers directly for more information or to take part. With a positive response informants were asked to supply their telephone number and best day and time to reach them. If they did not wish to be contacted, or if they did not feel they were the most informed about the decedent’s end-of-life care, they were asked to provide suggestions for an alternate person to whom an invitation may be sent. A follow-up reminder was sent to those who had not yet responded approximately three weeks following the initial mailing. Informants who agreed to participate were telephoned by one of two interviewers, trained extensively in approaching and asking questions of the bereaved, to make arrangements for the survey interview to take place and to confirm their knowledge of the decedent’s end-of-life care and interest in taking part [22].

Because the identification of potentially eligible informants and their initial contact originated from the Nova Scotia Vital Statistics (NSVS) office, the research team had no knowledge of who had been invited to take part in the study. Information pertaining to informants who did not respond or declined to participate was not permitted to be collected.

### Survey instrument

An adaption of the After-Death Bereaved Family Member Interview [10,25] was used. The instrument is reported to exhibit good validity and reliability [26] and targets decedent and family care experiences, needs and care preferences (wishes) for both the last month and last week of life. Key questions within the survey may be combined to create multiple patient-focused, family-centred domains and an overall satisfaction score. The tool has been used in the United States [10] and Canada [27] to identify areas of unmet need or concerns about care as perceived by family members in order to develop facility improvement strategies. Modifications for this study included additional questions related to location of care, location preferences, death awareness and the provision of provincially funded health services.

### Measures

#### Outcomes

Domains of patient-focused, family-centred care identified through the work of Teno et al. [17] were assessed in relation to the location where the majority of care occurred in the last 30 days of life. Six patient-focused, family-centred care domains elicited by the survey were created: 1) provision of desired physical comfort (pain, dyspnea) and emotional support (3 single items), 2) promotion of shared decision making (3 items), 3) treatment of the dying with respect (1 item), 4) attention to the needs of the family(caregivers) for information and knowing what to expect while the decedent was dying (2 composite scores: 3 items each), 5) attention to the needs of the family (caregivers) for emotional and spiritual support (7 items) and, 6) provision of coordination of care across care settings and health care providers (1 item). Assessment of overall satisfaction of patient-focused, family-centred care employed a single item based on a 0 to 10 point likert scale. Table 1 provides items associated with each domain and overall satisfaction.

**Table 1:** Patient-focused family-centred domain sample questions

**Domain**	**Response options**
*For all questions the informant was reminded to focus on the decedent’s last 30 days while he/she was at the location identified earlier through a series of questions, as to where the majority of care was provided.*	
*Physical comfort and emotional support*	
[Asked of informant’s if the decedent had experienced pain and were provided medications or treatment for their pain] …	
Did [DECEDENT] receive too much, too little, or just the right amount of medication for (his/her) pain?	[ ] Too much
	[ ] Too little
	[ ] Right amount
Similar questions were asked about help to treat dyspnea and support for feeling of anxiety and/or sadness (emotional support).	
*Promotion of shared decision making (among informants who had contact with the decedent’s doctor or nurse…)*	
Was there ever a problem understanding what any doctor or nurse was saying to you about what to expect from treatment?	[ ] Yes
	[ ] No
	[ ] No treatment
Was there ever a decision made about (his/her) care without enough input from (him/her) or (his/her) family?	[ ] Yes
	[ ] No
How much information did the doctors or nurses provide you about [DECEDENT’S] medical condition - would you say less information than was needed, just the right amount, or more than was needed?	[ ] Less than was needed
	[ ] Just the right amount
	[ ] More than was needed
*Treating the dying patient with respect*	
During those last 30 days how often was (he/she) treated with respect by those who were taking care of (him/her) - always, usually, sometimes, or never?	[ ] Always
	[ ] Usually
	[ ] Sometimes
	[ ] Never
*Attend to the needs of the family*	
a) Information needs	
At any time did you or your family receive any information about what to expect while (he/she) was dying? *(e.g. symptom relief (pain, breathing), emotions)*	[ ] Yes
	[ ] No
Would you have wanted some or more information about that?	[ ] Yes
	[ ] No
At any time did you or your family receive any information about what to do at the time of (his/her) death? *(process of who to call, contact …)*	[ ] Yes
	[ ] No
Would you have wanted some or more information about that?	[ ] Yes
	[ ] No
At any time during the time around [DECEDENT’S NAME] death, did you or your family receive any information about the medicines that would be used to manage (his/her) pain, shortness of breath, or other symptoms?	[ ] Yes
	[ ] No
Would you have wanted some or more information about the medicines?	[ ] Yes
	[ ] No
b) Caregiver skills – knowing what to expect as death approached	
How confident were you that you knew what to expect while [DECEDENT] was dying? Were you:	[ ] Very confident
	[ ] Fairly confident
	[ ] Not confident
How confident were you that you knew what to do at the time of death. Were you:	[ ] Very confident
	[ ] Fairly confident
	[ ] Not confident
How confident were you that you understood about the medicines that would be used to manage [his/her] pain, shortness of breath, or other symptoms. Were you:	[ ] Very confident
	[ ] Fairly confident
	[ ] Not confident
*Attend to emotional and spiritual needs of the family*	
During this time did someone talk with you about your religious or spiritual beliefs?	[ ] Yes
	[ ] No
If yes, was it done in a sensitive manner?	[ ] Yes
	[ ] No
Did you have as much contact of that kind as you wanted?	[ ] Yes
	[ ] No
How much support in dealing with your feelings about [DECEDENT’S] death did the doctors, nurses or other professional staff taking care of [him/her] provide you?	[ ] Less support than was needed
	[ ] Right amount
Did a doctor, nurse or other professional staff taking care of [DECEDENT] talk about how you might feel after [his/her] death?	[ ] Yes
	[ ] No
Would you have wanted them to?	[ ] Yes
	[ ] No
Was it done in a sensitive manner?	[ ] Yes
	[ ] No
*Provide coordination of care*	
During those last 30 days, was there any problem with doctors or nurses not knowing enough about [his/her] medical history to provide the best possible care?	[ ] Yes
	[ ] No
*Overall satisfaction with care*	
On a scale of 0 to 10, where 0 means the worst care possible and 10 means the best care possible, what number would you give the overall care that [DECEDENT] received during those last 30 days of life while being cared for at [LOCATION OF CARE]?	0 = worst care possible
	10 = best care possible

For each item a score of 1 was given if a service was not provided, was not provided fully, or not provided to the degree desired by the informant, or concerns were expressed (i.e. unmet need or had concerns) [25]. If the need was met or no concerns were indicated the item was scored as 0. For domains encompassing multiple items an additive domain composite score was created. All domains scores were dichotomized as having all needs met (or no concerns expressed) versus unmet need (had concerns). An overall satisfaction score of 10 was considered as all needs being met while a score of less than 10 was deemed as an indication of not being completely satisfied and the informant having at least some concerns or unmet need.

### Location of the majority of care

A series of questions targeting the location of care were asked early in the survey and asked the informant to recall where the decedent was located during each of the 30 days prior to death, how long they were in each location and if care was provided there. These questions and others asked may be viewed online [28]. The location with the greatest total number of days was considered where the patient received the majority of their care during the last 30 days of life. In the event of a tie, the location closest to the date of death was selected. Following confirmation of the location where the majority of the decedent’s care was provided during their last 30 days of life, informants were then repeatedly asked to focus their responses to the care provided to the decedent while in that identified location. Location of care was categorized as home (the decedent’s home, another’s home), hospital (acute/chronic care units), within a hospital-based palliative care unit (PCU) or in a long-term care facility (nursing homes, residential care) (LTC). The proportion of decedents who would have spent the majority of their last 30 days in a PCU was expected to be relatively small due to limited availability across the province. It was felt the PCU should be considered a separate location of care category because of the specialized care provided there and the relatively lower patient-staff ratios offered compared to regular hospital units, LTC and at home with home care. At the time of this study, free-standing hospice locations were not available in Nova Scotia.

### Covariates

Decedent sex, age, cause of death and location of death were obtained from the death certificate and confirmed during the interview. Informant information and additional decedent characteristics were collected during the survey process and included decedent marital status, living arrangements, whether they considered themselves a visible minority, education and income as well as informant sex, age, relationship to decedent, education, health status and awareness of approaching death.

### Study size

A priori power calculations taking into account past mortality statistics [2] and future analysis plans based on detecting a difference of 10% in a dichotomous outcome (needs met vs unmet) using a chi-square test conducted at the α = 0.05 level of significance [29] indicated a total of 1200 completed survey interviews were required.

### Analysis

Following descriptive statistics, Pearson chi-square tests and Fisher’s exact tests were used to first examine the association between location of care and decedent and informant characteristics and informant perceptions of the domains of care. This was followed by logistic regression techniques to assess the relationship between location of care and the perception of unmet need for each domain and overall satisfaction with and without adjustments for decedent and informant characteristics. Covariates used in all adjustments included decedent sex, age, cause of death, marital status, education, income, living alone, visible minority status, the informant’s relationship and informant awareness of the decedent’s approaching death.

## Results

Of the 5848 decedents identified by NSVS as potentially eligible for inclusion in the study, 289 invitations mailed to informants listed on the death certificate could not be delivered and 216 were found to be ineligible (due to informant death, hospitalization or poor cognition, no knowledge of care or no medical care provided to the decedent during the last 30 days of life). This resulted in 5343 potentially eligible informants. Consent to participate was provided by 1416 informants and 1358 survey interviews were conducted (response rate: 25.4%). For this analysis, 1316 survey interviews were used. The time required for each survey interview varied and depended largely on the care provided and the informants’ situation and knowledge (35 to 90 minutes). Ninety percent of interviews were conducted within one year of the decedent’s date of death (mean 9.7 months; SD 2.3 months).

### Decedent characteristics

Decedents tended to be female (51%), married (48%) and elderly with a mean age of 79 years (SD 13 years). Cancer was the most common cause of death (38%), followed by circulatory (24%), respiratory (10%) and nervous system disease (13%). Forty-nine percent of decedents were felt by their informant to be aware of their impeding death.

During their last 30 days of life, the majority of the decedent’s care was provided while they lived at home (39%), within LTC (29%), in the hospital (23%) or in a hospital-based PCU (9%). Fifty-three percent of decedents spent all of their last 30 days in one location, 39% in two locations, 6% three and 2% had four locations of care during their last 30 days of life. For 824 (63%) of decedents, the location of the majority of care during their last 30 days was the same as their place of death. All decedent characteristics differed significantly by their location of care (Table 2).

**Table 2:** Decedent and informant characteristics by location of care (n = 1316)

**Characteristic**		**Frequency (percent)**			
	**All**	**Location of care**			
		**Home**	**Hospital (acute/chronic care units)**	**Hospital palliative unit**	**Long-term care**
***Decedents*** (n)	1316 (100)	514 (39.1)	297 (22.6)	120 (9.1)	385 (29.3)
**Sex**^§^					
Female	675 (51.3)	226 (44.0)	135 (45.5)	60 (50.0)	254 (66.0)
**Age group** (years)^§^					
19-64	189 (14.4)	121 (23.5)	40 (13.5)	22 (18.3)	6 (1.6)
65-84	591 (44.9)	256 (49.8)	138 (46.5)	64 (53.3)	133 (34.6)
85+	536 (40.7)	137 (26.7)	119 (40.1)	34 (28.3)	246 (63.9)
Mean age (std)^§^	79.1 (12.8)	74.8 (13.1)	78.3 (12.8)	75.5 (12.6)	86.4 (8.6)
Median (range)	81 (19–107)	76.5 (19–103)	81 (24–107)	78 (43–98)	87 (28–101)
**Cause of death**^§^					
Cancer	501 (38.1)	286 (55.8)	93 (31.3)	84 (70.0)	38 (9.9)
Circulatory system disease	319 (24.2)	105 (20.5)	87 (29.3)	18 (15.0)	109 (28.3)
Respiratory system disease	129 (9.8)	36 (7.0)	35 (11.8)	7 (5.8)	51 (13.3)
Nervous system/mental and behavioural disorders	170 (12.9)	30 (5.8)	18 (6.1)	5 (4.2)	117 (30.4)
Other causes	197 (15.0)	57 (11.1)	64 (21.6)	6 (5.0)	70 (18.2)
**Cancer cause of death**^§^					
Cancer	501 (38.1)	286 (55.8)	93 (31.4)	84 (70.0)	38 (9.9)
Non-cancer	813 (61.9)	227 (44.3)	203 (68.6)	36 (30.0)	347 (90.1)
**Marital status**^§^					
Married	629 (47.8)	321 (62.5)	142 (47.8)	66 (55.0)	100 (26.0)
Divorced/separated	92 (7.0)	30 (5.8)	29 (9.8)	9 (7.5)	24 (6.2)
Never married	80 (6.1)	22 (4.3)	22 (7.4)	13 (10.8)	23 (6.0)
Widowed	515 (39.1)	141 (27.4)	104 (35.0)	32 (26.7)	238 (61.8)
**Lived alone**^§^					
Yes	229 (17.4)	100 (19.5)	80 (26.9)	32 (26.7)	17 (4.4)
Perceived themselves as a visible minority^*^					
Yes	94 (7.2)	30 (5.9)	32 (10.8)	11 (9.2)	21 (5.5)
No	1206 (92.0)	479 (93.9)	261 (88.2)	107 (89.2)	359 (93.3)
Don’t know	11 (0.8)	1 (0.2)	3 (1.0)	2 (1.7)	5 (1.3)
**Education** (highest level completed)^§^					
Completed postsecondary	340 (25.8)	170 (33.1)	71 (23.9)	32 (26.7)	67 (17.4)
Some postsecondary	166 (12.6)	71 (13.8)	39 (13.1)	16 (13.3)	40 (10.4)
High school	217 (16.5)	82 (16.0)	47 (15.8)	21 (17.5)	67 (17.4)
Less than high school	562 (42.7)	179 (34.8)	135 (45.5)	49 (40.8)	199 (51.7)
Don’t know	31 (2.4)	12 (2.3)	5 (1.7)	2 (1.7)	12 (3.1)
**Income** (dollars)^§^					
Less than 20,000	319 (24.2)	76 (14.8)	74 (24.9)	22 (18.3)	147 (38.2)
20,000 – 29,999	291 (22.1)	100 (19.5)	62 (20.9)	33 (27.5)	96 (24.9)
30,000 – 49,999	230 (17.5)	97 (18.9)	58 (19.5)	24 (20.0)	51 (13.3)
Greater than 50,000	312 (23.7)	179 (34.8)	66 (22.2)	25 (20.8)	42 (10.9)
Don’t know/refused	164 (12.5)	62 (12.1)	37 (12.5)	16 (13.3)	49 (12.7)
**Aware of own approaching death**^§^					
Yes	649 (49.3)	320 (62.3)	131 (44.1)	66 (71.7)	112 (29.1)
No	466 (35.4)	133 (25.9)	122 (41.1)	16 (13.3)	195 (50.7)
Not sure	201 (15.3)	61 (11.9)	44 (14.8)	18 (15.0)	78 (20.3)
***Informant***					
**Sex**^†^					
Female	926 (70.4)	382 (74.3)	217 (73.1)	75 (62.5)	252 (65.5)
**Age group** (years)					
19-64	717 (54.5)	282 (55.0)	167 (56.2)	61 (50.8)	207 (53.8)
65-84	542 (41.2)	218 (42.5)	114 (38.4)	53 (44.2)	157 (40.8)
85+	56 (4.3)	13 (2.5)	16 (5.4)	6 (5.0)	21 (5.5)
Mean age (std)	63.9 (11.4)	63.3 (11.5)	64.1 (11.5)	63.6 (12.7)	64.8 (10.7)
Median (range)	63 (27–96)	63 (29–92)	63 (27–96)	64 (29–91)	64 (32–93)
**Relationship to decedent**^§^					
Spouse/partner	473 (35.9)	262 (51.0)	98 (33.0)	49 (40.8)	64 (16.6)
Their child	638 (48.5)	200 (38.9)	139 (46.8)	44 (36.7)	255 (66.2)
Other	205 (15.6)	52 (10.1)	60 (20.2)	27 (22.5)	66 (17.1)
**Education** (highest level completed)					
Completed postsecondary	657 (49.9)	273 (53.1)	140 (47.1)	55 (45.8)	189 (49.1)
Some postsecondary	247 (18.8)	90 (17.5)	66 (22.2)	24 (20.0)	67 (17.4)
High school	234 (17.8)	83 (16.2)	49 (16.5)	20 (16.7)	82 (21.3)
Less than high school	175 (13.3)	65 (12.7)	42 (14.1)	21 (17.5)	47 (12.2)
Don’t know/refused	3 (0.2)	3 (0.6)	0	0	0
**Health status**					
Excellent	289 (22.0)	104 (20.4)	67 (22.0)	27 (22.7)	91 (23.6)
Very good	525 (40.0)	195 (38.2)	117 (39.4)	48 (40.3)	165 (42.9)
Good	344 (26.2)	146 (28.6)	73 (24.6)	30 (25.2)	95 (24.7)
Fair	114 (8.7)	47 (9.2)	30 (10.1)	7 (5.9)	30 (7.8)
Poor	40 (3.1)	19 (3.7)	10 (3.4)	7 (5.9)	4 (1.0)
**Aware of decedent did not have long to live**^§^					
Yes	734 (55.8)	295 (57.4)	145 (48.8)	90 (75.0)	204 (53.0)
No	339 (25.8)	126 (24.5)	91 (30.6)	13 (10.8)	109 (28.3)
Not sure	243 (18.5)	93 (18.1)	61 (20.5)	17 (14.2)	72 (18.7)

Differences were assessed using Pearson chi square and Fisher’s exact tests of association for categorical variables; one-way analysis of variance for continuous variables: ^*^p < 0.05; ^†^p < 0.01; ^§^p < 0.0001.

### Informant characteristics

Informants were predominately female (70%), middle-aged (mean age 63.9 years; SD 11.4), highly educated (50% completed postsecondary education) and considered themselves in excellent or very good health (62%). They tended to be either the decedent’s child (49%) or their spouse/partner (36%). During the decedent’s last 30 days of life, 56% of informants were aware that the decedent did not have long to live. Informants differed significantly by location of care with respect to this awareness of approaching death, sex and their relationship to the decedent (Table 2).

### Domain and satisfaction scores

**Figure 1:**
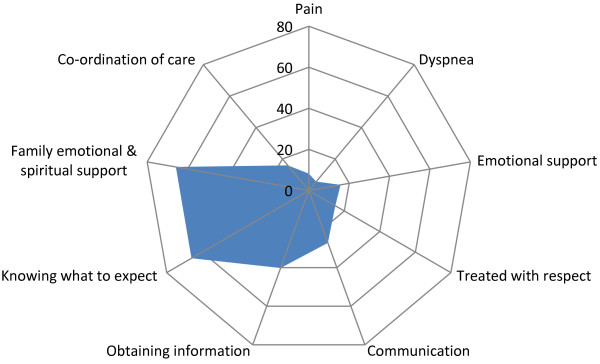
Proportion of informants expressing unmet need/concerns within each domain (n = 1316).

Results indicating the proportion of unmet need or concerns perceived by the informant for each domain and overall satisfaction are illustrated in Figure 1 and summarized for all and by location of the majority of care in the last 30 days in Table 3. Among all informants, the degree of unmet need ranged widely from 5.6% reporting a lack of desired help with dyspnea to 66% expressing concerns about their confidence in knowing what to expect close to the decedent’s death and for unmet support provided for the emotional and spiritual needs of the family. Unadjusted analyses indicate significant differences by location of care within most domains. Exceptions were the provision of desired help for decedent pain, where relatively low unmet need in all locations was indicated, and support for the emotional and spiritual needs of the family, where a large degree of unmet need was expressed across all. Although the mean score for overall satisfaction with patient-focused, family-centred care proved relatively high (mean = 8.7; SD 1.8), 57% were not completely satisfied with the overall care provided to the decedent, indicating at least some unmet need or concerns.

**Table 3:** Informant perceptions of unmet need/concerns by the location where the majority of care was provided during last 30 days of life

**Domain**	**Perception of unmet need/concerns**				
	**Frequency (percent)**				
	**All**	**Home**	**Hospital (acute/chronic care)**	**Hospital- palliative unit**	**Long-term care**
Physical comfort and emotions:					
Decedent did not receive desired help for:					
Pain	108 (8.2)	46 (9.0)	23 (7.7)	8 (6.7)	31 (8.1)
Dyspnea^§^	74 (5.6)	49 (9.5)	12 (4.0)	0 (0)	13 (3.4)
Emotional support^†^	206 (15.7)	84 (16.3)	63 (21.2)	11 (9.2)	48 (12.5)
Promote shared decision making^§^:					
Concerns with communication	332 (27.2)	106 (23.8)	110 (37.8)	31 (26.1)	85 (23.3)
Concerns with the decedent being treated with respect^§^	194 (14.9)	41 (8.1)	71 (24.4)	19 (15.8)	63 (16.5)
Attend to the needs of the family:					
a) Unmet need in obtaining information^‡^	525 (40.2)	220 (43.1)	137 (46.8)	37 (30.8)	131 (34.3)
b) Concerns with knowing what to expect^†^	862 (66.0)	346 (67.7)	211 (72.0)	80 (66.7)	225 (58.9)
Concerns about support for the emotional and spiritual needs of the family	860 (65.8)	338 (66.4)	196 (66.4)	77 (64.2)	249 (65.0)
Coordination of care concerns^†^	202 (15.9)	81 (16.2)	61 (21.7)	11 (9.2)	49 (13.2)
Overall satisfaction^‡^:					
Not completely satisfied	729 (57.0)	256 (51.1)	190 (66.4)	64 (54.2)	219 (58.6)

Differences were assessed using Pearson chi square and Fishers exact tests of association for categorical variables: ^†^p < 0.01; ^‡^p < 0.001; ^§^p < 0.0001.

### Multivariable adjustments

Table 4 reports all results, both unadjusted and adjusted for the odds of experiencing unmet need or concerns for each domain of care examined. Following adjustments for decedent and informant characteristics, patients spending the majority of their last month in hospital (adjusted Odds Ratio [AOR]:0.3; 95% Confidence Interval [CI]: 0.1-0.6) and LTC (AOR:0.2; 95% CI: 0.1-0.5) were perceived by the informant as experiencing less unmet need for help with dyspnea than decedents at home. Within the PCU, decedents either did not experience dyspnea or all breathing related needs were felt to be fully met. Compared to decedents cared for in the home, twice as many informants expressed concerns with shared decision making and communication when the majority of care was provided in hospital (AOR:2.0; 95% CI: 1.4-2.8). The decedent being treated with respect was a greater concern when care was provided primarily in hospital (AOR:3.2; 95% CI: 2.1-5.0), in a PCU (AOR:2.1; 95% CI: 1.1-3.9) or in LTC (AOR:2.3; 95% CI: 1.4-3.8) compared to home. Compared to home, less unmet need in obtaining information was perceived by informants of decedents in a PCU (AOR:0.6; 95% CI: 0.4-1.0) and in LTC (AOR:0.7;95% CI: 0.5-1.0). Informants of decedents in LTC were less likely to report concerns with knowing what to expect or what to do at time of death (AOR:0.7; 95% CI: 0.5-0.9) and for concerns about family support for emotional and spiritual (AOR:0.7; 95% CI: 0.5-1.0) than those cared for at home. Following adjustments less concern with coordination of care was perceived if the majority of care was provided in a PCU (AOR:0.5; 95% CI: 0.2-1.0). Compared to home, informants of those in hospital were 60% more likely not to be completely satisfied with the overall patient-focused family-centred care provided (AOR:1.6; 95% CI: 1.2-2.3). No statistically significant differences were evident in the provision of help for decedent pain or emotional support before or following adjustments by location of care.

**Table 4:** Unadjusted and adjusted odds of experiencing unmet need or concerns

	**Location of care**						
	**Unadjusted (Unadj) and Adjusted**^**1 **^**(Adj) Odds ratio (95% confidence interval)**						
**Outcomes:**	**Home**	**Hospital (acutechronic care)**		**Hospital palliative unit**		**Long-term care**	
Decedent did not receive the desired help for:		Unadj	Adj	Unadj	Adj	Unadj	Adj
Pain	Referent	0.9 (0.5-1.5)	0.8 (0.5-1.5)	0.8 (0.3-1.7)	0.8 (0.4-1.9)	0.9 (0.5-1.4)	1.2 (0.7-2.2)
Dyspnea^2^	Referent	0.4 (0.2-0.7)	0.3 (0.1-0.6)	100% had their needs met		0.3 (0.2-0.6)	0.2 (0.1-0.5)
Emotional support	Referent	1.3 (0.9-1.9)	1.1 (0.7-1.7)	0.6 (0.3-1.1)	0.6 (0.3-1.1)	0.8 (0.5-1.1)	0.7 (0.4-1.1)
Shared decision making. Informant had concerns with communication	Referent	1.9 (1.4-2.6)	2.0 (1.4-2.8)	1.1 (0.7-1.8)	1.3 (0.8-2.1)	1.0 (0.7-1.4)	1.2 (0.8-1.8)
Concerns with the decedent being treated with respect	Referent	3.7 (2.4-5.6)	3.2 (2.1-5.0)	2.0 (1.1-3.6)	2.1 (1.1-3.9)	2.2 (1.5-3.4)	2.3 (1.4-3.8)
Attend to the needs of the family							
a) Unmet need in obtaining information	Referent	1.1 (0.9-1.5)	1.0 (0.8-1.4)	0.6 (0.4-0.9)	0.6 (0.4-1.0)	0.7 (0.5-0.9)	0.7 (0.5-1.0)
b) Concerns with knowing what to expect	Referent	1.2 (0.8-1.6)	1.1 (0.8-1.5)	1.0 (0.6-1.5)	1.1 (0.7-1.7)	0.6 (0.5-0.9)	0.7 (0.5-0.9)
Concerns about support for the emotional and spiritual needs of the family	Referent	1.0 (0.7-1.4)	0.9 (0.6-1.2)	1.0 (0.7-1.5)	1.0 (0.6-1.6)	1.0 (0.8-1.4)	0.7 (0.5-1.0)
Coordination of care concerns	Referent	1.4 (0.9-2.0)	1.4 (0.9-2.0)	0.5 (0.2-1.0)	0.5 (0.2-1.0)	0.8 (0.5-1.1)	1.0 (0.6-1.6)
Not completely satisfied with overall care	Referent	1.8 (1.4-2.5)	1.6 (1.2-2.3)	1.1 (0.7-1.6)	1.1 (0.7-1.7)	1.3 (1.0-1.8)	1.2 (0.8-1.6)

^1^Multivariate logistic regressions adjusted for decedent sex, age, cause of death, marital status, education, income, living alone, visible minority, informant relationship status and informant awareness of the decedent’s approaching death.

^2^No concerns were expressed by informants of all decedents who spent the majority of their care in a hospital palliative unit during the last 30 days with respect to dyspnea care.

## Discussion

### Key findings

To our knowledge this is the first population-based mortality follow-back survey undertaken in Canada. Although others in Canada have recently reported on family members’ perceptions of care received at the end of life, those participants were primarily associated with patients within institutions, special care programs or organizations [27,30-33]. Internationally, this is also one of the few population-based initiatives where a) questions were focused on the decedent’s primary location of care during the last 30 days of life, not the final location of death, and b) there was sufficient analytic capability to apply multivariable adjustments in the examination of differences between locations.

Although some differences by location of the majority of care during the last 30 days of life were evident, no one location stood out as being exceptionally better or worse in terms of reported unmet need or concerns. Multivariable adjustments tended to exhibit minor effects on statistical estimates. It is encouraging to report that the overall score associated with satisfaction with patient-focused, family-centred care was perceived by the bereaved as being quite good. However, less than half felt ‘the best care possible’ was provided during the last month of life. Significant differences in this overall satisfaction were perceived by the location of the majority of care during the last 30 days of life. Compared to home, greater dissatisfaction with overall patient-focused, family-centred care provided was associated with the acute care hospital setting as was greater concern with the domain of communication. Less dissatisfaction and fewer concerns in general were expressed about care provided in LTC facilities and within specialized hospital-based PCUs. Concerns about the decedent being treated with respect were much higher in all locations compared to home.

Somewhat surprisingly to us, was our important finding that the perception of care provided to adult Nova Scotians tended to vary more widely across targeted patient-focused, family-centred domains than between locations of care. Across all locations where the majority of care was provided during the last 30 days of life, the degree of unmet need or concerns associated with desired care provided for decedent physical comfort (pain, dyspnea) and emotional support were perceived to be relatively low while moderate concerns were expressed by all with coordination of care and treatment with respect. One example of variation by location was dyspnea, with higher unmet need in the home setting. This is not surprising given that hospital settings have immediate response health professionals available (such as a respiratory therapist) that are not available in homes. In contrast, across all locations, informants identified a very high degree of unmet need or concern with respect to the family knowing what to expect as death approached, obtaining information and family support for emotional and spiritual needs.

### Limitations

A number of limitations are associated with this study. Our goal was to target a maximal adult population [21] of all who had died of advanced disease during the two year study period. However, more than half of deaths were not yet confirmed and therefore not available in the NSVS database within the desired three to seven months following the date of death. This was due primarily to the length of the National death certificate clearance process. As such many potentially eligible death certificates were not identified and thus their informants not invited to participate. Although possible, in discussion with NSVS staff, we do not believe that death certificates unavailable for identification in each selection wave would have resulted in systematic selection bias.

Challenges associated with the identification of eligible deaths, the inability to directly contact potential informants, not knowing whether the intended informant received the mailed invitation or not, and the highly emotional topic all combined to result in a response rate of 25%. In a previously published article we address these challenges and resolution strategies [22] in taking a population approach to assess care experiences. Although disappointing, the number of informants participating in the study was large and surpassed the targeted sample size. Study decedents proved to be relatively representative of Statistics Canada death statistics for Nova Scotia [2] with respect to the population distribution of causes of death, age and sex. However, informants taking part were self-selected and we do not know how representative their perceived experience of care and unmet need was relative to those who did not respond. Despite explicitly asking all with various experiences to take part (good or bad), it is possible that a greater proportion of informants who perceived better care being provided to their decedent participated which could potentially result in an underestimation of unmet need. It is also possible that the reverse situation could have occurred. In reviewing our results, they do indicate a comparatively low perception of unmet need with respect to symptom control compared to what some have reported [10,23,29,30]. In contrast, there was much unmet need expressed within specific domains such as obtaining information, knowing what to expect and support for the emotional and spiritual needs of the family, all supporting what others have found [10,23,31,32,34].

A limitation of the mortality follow-back survey design in general relates to the validity of participant’s responses as proxies for the decedent’s experience. Research suggests family members’/caregivers’ responses show moderate to substantial agreement with patients’ responses regarding objective items (such as observable symptoms, patient functioning, use of services), but less agreement on subjective items (such as pain, anxiety and spiritual concerns) [34-37]. Caregiver assessments of pain tend to be rated moderately higher than patients’ assessments [36,37], polarize toward the scale’s extremes after death compared to before death [38], and vary with the timing of the interview and the reference period for the item compounding the problem of comparisons across regions [33,39]. In this study, with the exception of symptom control, the focus was primarily on the needs and experiences of the informant and/or the family unit and their perceptions of patient-focused family-centred care. These perspectives are integral to understanding quality of life and quality of care at end of life and have their own validity [27,37,38,40].

We asked informants to focus on the mutually agreed upon location identified as where the majority of care was provided during the last 30 days of life. It is possible that at times, some informants may have experienced difficulties in focusing on this one identified location and/or the 30 day time frame. However, great efforts were made when presenting each question to the informant to sensitively remind them to focus on the mutually agreed upon location and timeframe.

Finally, the use of a survey design in itself has limitations. Most responses are fit into predefined categories that may not capture individual experiences or provide enough detail to understand the circumstances and how needs are not being met. Despite this, survey designs are important in the provision of a population perspective and may be used to aid identification of what works best in the healthcare system and where opportunities for improvement lie. Qualitative research may then be conducted to follow-up on key survey perspectives.

### Interpretation

Some of these results support what others have reported, while others differ. However, direct comparisons may be difficult to make given health care systems across different countries and even within the same country differ, as do the care processes within and societal values. As such we can look at the results reported by each but, when drawing conclusions, readers must consider their own settings. We must also acknowledge that informants’ satisfaction with care does not necessarily correspond to independent outcome measures of care such as symptom control [41].

Our finding of less satisfaction with an acute care hospital setting echo recent United Kingdom [16] national results where hospital care ratings associated with location of death tended to be lower. In contrast our results differ somewhat from that reported by both Teno (US) [10] and Gallagher (Canada) [23], who, using the same survey tool, reported greater dissatisfaction associated with care provided by nursing homes and residential care. Although these variations in findings may be a reflection of how healthcare is organized across settings and within different countries, it is important to note that the time frame under examination differs. These latter two studies both focused on the location of care during the last week or days of life whereas our current focus was on the location where the majority of care was provided over the last 30 days. All studies confirm better care experiences with hospice involvement or in a PCU, a finding not unexpected given the specialized palliative care provided there.

In Nova Scotia, most informants felt the right amount of help was provided for decedent pain, dyspnea and, to a lesser degree, emotional support with little unmet need expressed. However many studies report much higher proportions of unmet need or not enough support for these conditions [10,33,42,43]. For instance in Germany [44], 33% of informants felt pain was not treated sufficiently during the last four weeks of life. Similarly Australian researchers report approximately 30% [39] of family members felt they did not receive enough support from health services for pain, dyspnea, anxiety or depression. Using the same survey tool as this study, Teno’s work in the US [10] and Gallagher’s Canadian study [27] also report a similar higher degree of family perception of unmet need for pain, dyspnea and emotional support. Again it is important to note that each of these latter two studies focused only on the last 48 hours of life, a time period where symptoms and the response needed often escalate. Information focused on these very last days was collected as part of this Nova Scotia study and will be a topic of a future article.

Adequate emotional support is one of the key issues identified by patients and their families as requiring improvement in Canada in order to increase the satisfaction of care for persons at the end of life [45]. The large degree of unmet need of emotional and spiritual support for the family perceived by informants participating in this study (Table 3), and among others internationally [10,27,39,44,46,47], confirms this perception is widespread. Research suggests healthcare providers and family members may perceive emotional and spiritual support differently. Health service providers tend to view the offer of support in the form of relational and active-based care whereas family and caregivers view supportive care as having conversations and shows of regard [35,48].

Informants identified various forms of communication, such as decision-making involvement, medication knowledge and the provision of information about what to expect and do at the time of death as being largely unmet (Table 3). Although many were provided information, over half of informants did not feel prepared or felt knowledgeable about the medications used for symptom management. Similar to the need for emotional support, the lack of adequate communication during the time when death was approaching has been documented internationally [10,27,34,44,47] and identified by family members and patients as a priority area for the improvement [34,45]. Recently, the Canadian Hospice Palliative Care Association has been working with other organizations to address this problem through its ‘Speak Up’ campaign to improve advance care planning [36]. Other countries have also been developing programs to address this need.

The consistent perceived lack of emotional support and communication concerns across all care locations clearly signal the need for health service providers and stakeholders to attend to these important elements of care for both patients and their families. The need for specific programs or targeted interventions to enhance these skills may be required [44,45].

We do not find it surprising that informants in this study expressed greater concerns with the decedent being treated with respect in all locations other than home. Most informants were close family members who could potentially hold mixed feelings about relinquishing much of the care and responsibility of their loved ones to others and/or considered their treatment of the decedent at home as being performed with the highest of respect. Regardless, this finding needs to be considered in our care delivery in all settings.

Overall these findings suggest there is room for improvement in patient-focused and family-centred care across all care settings. Variations in unmet needs may provide an opportunity for care settings to learn from each other. For example, findings confirm attention to home-based treatment for dyspnea is needed suggesting people operating in the home setting could learn from best practice treatment of dyspnea in other settings. In return, hospital and other personnel can hopefully improve in their respect of persons at end of life by listening to and learning from families. Processes for success in coordinating care and providing information in a hospital-based PCU can hopefully be transferred to the home, LTC and other hospital settings. Persons operating in LTC settings may have strategies for others in alternate settings on attending to the needs of families’ knowledge and the provision of family support.

In examining the variations in unmet need by care location, consideration must be given to the policy and financial support to each. For example, in-hospital PCUs are richly resourced, particularly in human resources, compared to the long-term care setting in our province. Strategies to influence policy or integrate care resources across location settings may help improve outcomes (e.g. specialized palliative care teams who are able to provide consulting services in the LTC setting).

## Conclusion

Overall satisfaction with patient-focused family-centred care provided during the last 30 days of life as reported by bereaved family members of decedents was high in Nova Scotia with the least satisfaction being expressed for the acute care hospital setting. Although no one location stood out as exceptionally different in terms of perceived unmet need or concerns, some variations by the location of the majority of care during decedent’s last 30 days were identified. Key findings indicated communication in various forms and emotional and spiritual support for the family were viewed as consistently lacking. Although some design limitations were identified, the strengths of this study have culminated in the provision of a ‘window’ into the care received at the end of life from the family’s perspective which are population-based and not limited to a single cause of death or location of care. These results improve our understanding of the extent of unmet need or concerns with care provided to adults who die in Nova Scotia from the family’s perspective and provide evidence of where improvements in patient-focused and family-centred care may first be targeted in order to impact quality care at end of life. By combining the results of this survey with other evaluations of end-of-life care we can continue to improve.

## Abbreviations

UK: United Kingdom; NSVS: Nova Scotia Vital Statistics; PCU: Palliative care unit; LTC: Long-term care; AOR: Adjusted odds ratio; CI: Confidence interval.

## Competing interests

The authors report no conflicts of interest.

## Authors’ contributions

FB conceived the study, participated in the design and coordination, helped to draft and finalize the manuscript. BL participated in the conceptualization and design of the study, acted as coordinator, performed the statistical analyses and helped to draft and finalize the manuscript. GJ participated in the conceptualization of the project, aided with the interpretation of results and helped to finalize the manuscript. YA, PM, EG and GF participated in the conceptualization of the project and helped to finalize the manuscript. All authors read and approved the final manuscript.

## Pre-publication history

The pre-publication history for this paper can be accessed here:

http://www.biomedcentral.com/1472-684X/13/25/prepub
